# Presentation Modes, Anticipation, and Penetrance: A Case Series of Huntington’s Disease

**DOI:** 10.7759/cureus.94101

**Published:** 2025-10-08

**Authors:** Towhida Sumaiya, Rezvey Sultana, Mofazzal Hossain Mridul

**Affiliations:** 1 Medicine, Dhaka Medical College Hospital, Dhaka, BGD; 2 Neurology, Dhaka Medical College Hospital, Dhaka, BGD; 3 Medicine, Poole Hospital, Poole, GBR

**Keywords:** anticipation, genetic variation, htt gene, huntington’s disease, penetrance

## Abstract

Huntington’s disease (HD) is an inherited, potentially incurable neurodegenerative disease. It typically presents as a triad of progressive psychiatric, cognitive, and motor symptoms and shows significant anticipation and penetrance. It is caused by trinucleotide CAG (cytosine, adenine, guanine) repeat expansion in the *Huntington* (*HTT*) gene. It exhibits significant anticipation, earlier onset in successive generations within a pedigree, which is caused by the instability of the *HTT* gene, along with a further increase in its length in subsequent generations. Reduced penetrance alleles of HD are relatively common in the general population. The threshold for disease is 35, with complete penetrance above 39 and incomplete penetrance for 36, 37, and 38 repeats. A series of three cases of HD have been discussed here, along with their pattern of presentation, anticipation, and genetic variation.

## Introduction

Huntington’s disease (HD) is a chronic neurodegenerative disorder characterized by the triad of clinical hallmarks, namely, chorea, cognitive impairment, and behavioral disorders [1,2]. The disease typically presents symptoms around the age of 40 years, but in 5-10% of cases, it begins before the age of 20 years [3]. The condition is inherited in an autosomal dominant manner and occurs in all racial groups, with worldwide morbidity of 6-14 per 100,000 individuals [4,5]. It usually presents with neurological and psychiatric symptoms, including movement disorders (most commonly chorea), dementia, depression, and other cognitive disturbances [6]. HD causes progressive degeneration of parts of the basal ganglia, specifically the caudate nucleus and the putamen [7,8].

The genetic basis involves unstable CAG (cytosine, adenine, guanine) repeat expansion in the *Huntington* (*HTT*) gene [3,9,10]. The resulting HTT protein causes dysfunction and death of neurons, with full penetrance of HD resulting from more than 40 CAG repeats, and partial penetrance can be seen between 36 and 39 CAG repeats [10]. It shows significant anticipation due to the instability of the *HTT* gene, along with a further increase in its length in subsequent generations. Anticipation simply means that the severity of illness in the affected child is greater than that of the affected parent [10]. Characteristic features may also be seen on MRI. There is no curative treatment available for HD, and management relies upon symptomatic and supportive measures [9].

## Case presentation

Case 1

A 24-year-old apparently well man presented to us with progressive abnormal behavior and restlessness for the last three years. Two years later, he developed choreiform movement of all four limbs, which worsened over time and incapacitated his day-to-day activities. Additionally, he experienced increasing forgetfulness, struggled with daily common tasks, and exhibited emotional instability.

**Figure 1:**
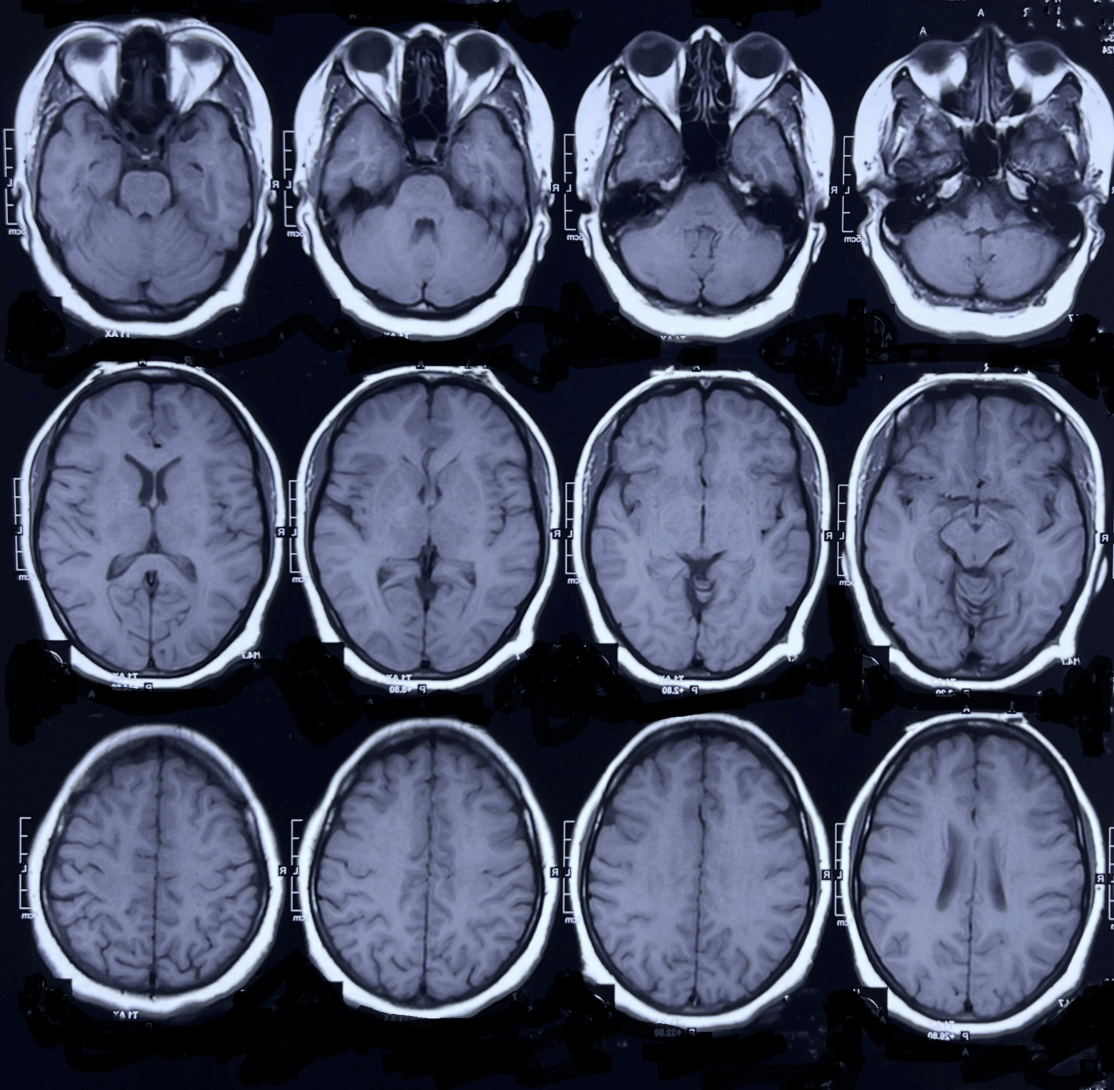
Normal MRI of of the brain (axial view).

On neurological examinations, he was found to be irritable and aggressive, with difficulty concentrating. Along with bradykinesia, rigidity and some gait difficulty were also noted. His score on the Mini-Mental State Examination (MMSE) was 18. Involuntary movement in the form of chorea was noted on the limbs, tongue, and face. Other neurological findings were unremarkable. All sets of investigations, including imaging, were performed and came back normal (Figure 1).

Consequently, he was finally diagnosed with HD by genetic analysis, which revealed 18 CAG repeats in one allele and 51 CAG trinucleotide repeats in the other allele of the *HTT* gene on chromosome 4, thus confirming the diagnosis of HD. In his family, his paternal grandmother and paternal uncle both showed abnormal movement of the limbs and forgetfulness at the ages of 35 years and 28 years, respectively. They remained undiagnosed and died at an early age. This family clearly showed anticipation.

Case 2

A 54-year-old man presented to the movement disorder outpatient department with a disabling tremor and abnormal movements of his limbs for five years. Along with these symptoms, he also had aggressive behavior and forgetfulness. He also had a history of falls followed by a subdural hematoma before admission.

**Figure 2:**
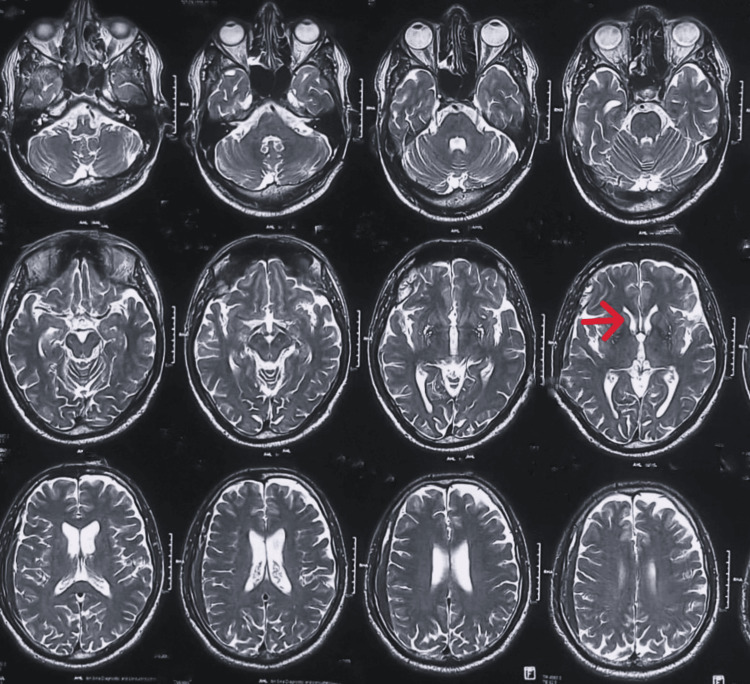
MRI of the brain (axial view) showing bilateral caudate nuclei atrophy.

On physical examinations, prominent chorea was noted in the extremities, tongue, and face, along with tremor both in rest and posture. Other systemic examinations, including neurological findings, were unremarkable except for the MMSE score of 20. Routine investigation, along with special tests for Wilson’s disease, was normal. MRI of the brain showed bilateral atrophy of the caudate nuclei, along with a right subdural hematoma (Figure 2).

With high clinical suspicion, genetic analysis was performed, and he was diagnosed with HD. The results of diagnostic testing revealed a normal allele with 19 CAG repeats and an expanded as well as unstable allele with 44 CAG repeats on chromosome 4. In his family, both his elder brother and sister had the same type of symptoms but died before a diagnosis. Now his two nephews are suffering from HD. In this family, all members showed symptoms after the age of 35 years. Anticipation was not evident.

Case 3

In this scenario, two brothers, 22 and 30 years old, visited our outpatient department with the same set of symptoms. Both presented with abnormal movements of the limbs for a similar duration of two years, but no cognitive impairment or psychotic symptoms. Initial neurological examinations were normal except for signs of chorea and mild tremor.

On a detailed history taking, we found out that their mother died with similar symptoms. Among the six siblings, all had features of HD except the eldest one. We performed genetic analysis of one of them and found a normal allele with 17 CAG repeats and an expanded and unstable allele with 45 CAG repeats in exon 4 of *HTT*. Finally, he was diagnosed with HD. This family showed clear anticipation as the younger ones were affected at an early age; however, they did not have a full triad of symptoms. None of their children has been affected till now.

## Discussion

HD is an inherited, potentially incurable neurodegenerative disease. It typically presents as a triad of progressive psychiatric, cognitive, and motor symptoms, as well as showing significant anticipation and penetrance [1,2]. Table 1 presents a brief summary of the three cases described here.

**Table 1:** Clinical, imaging, and genetic spectrum of Huntington’s disease patients. CAG: cytosine-adenosine-guanine; MMSE: Mini-Mental State Examination

Particulars	Case 1	Case 2	Case 3
Patient age	24	54	22
Gender	Male	Male	Male
Age of onset (years)	21	49	20
Disease duration (years)	3	5	2
Family history	Positive	Positive	Positive
Clinical features	Abnormal behavior, restlessness, chorea, cognitive impairment, Parkinsonism, and an MMSE score of 18	Aggressive behavior, cognitive impairment, tremors, chorea, and an MMSE score of 20	Chorea, mild tremors, and an MMSE score of 28
Triad of symptoms	Present	Present	Absent
Anticipation	Present	Absent	Present
Penetrance	Complete	Complete	Complete
Imaging of the brain	Normal	Head of bilateral caudate atrophy	Normal
Genetic study	51 CAG trinucleotide repeats	44 CAG trinucleotide repeats	45 CAG trinucleotide repeats

Mode of presentation

In the prodrome of HD, people may experience mild alterations in their personality, motor abilities, and cognitive abilities. Before the clinical onset of manifest HD, these modest changes may start to appear as early as 15 to 20 years [10-12]. The average age of onset for HD is around 45 years [1]. Approximately two-thirds of those affected first develop neurologic symptoms, while others have psychological problems. In its initial stages, the disease may present with brief changes in eye movement, mild incoordination, subtle involuntary movements, difficulties with planning, and mood changes [1,11]. Most affected individuals are able to perform the majority of their regular activities [1,11].

About 25% of HD patients experience delayed onset until after the age of 50 years, and some do so until the age of 70 years. These people experience chorea, dysphagia, and abnormalities in gait, but their symptoms are more prolonged and benign than those of a typical individual [1,11].

In the subsequent stage, chorea becomes more pronounced, voluntary motor control declines, and dysarthria and dysphagia progress [3,11]. Many individuals are forced to leave employment and increasingly depend on others for support, although a degree of personal independence is often retained. Functional impairment is considerable at this stage, and may be accompanied by episodes of aggression and socially inappropriate behavior [3,9,11].

In the late stages of HD, motor impairment becomes severe, and the individual is frequently completely reliant, mute, and incontinent. The median survival period from onset is 15 to 18 years (range: 5 to >25 years). The average age of death is 54 to 55 years [1].

Penetrance

Alleles containing 36-39 CAG repeats are thought to cause HD. However, their penetrance is not full. People with CAG repeats in this range who are elderly and asymptomatic are not uncommon [13]. More than 95% of alleles have the common [(CAG)n-CAA-CAG] interrupted repeat, which increases disease risk, while only 1% of alleles have the rare [(CAG)n] uninterrupted repeat [14-16].

The loss of interruption variant is characterized by the absence of the usual CAA interruption within the CAG repeat tract of the *HTT* gene. This results in an uninterrupted CAG sequence, which increases repeat instability and is generally associated with a higher risk of disease. Alleles are fully penetrant if they have more than 40 CAG repeats. There have been no reports of elderly individuals who are asymptomatic and have alleles of more than 40 CAG repeats [15,16].

Anticipation

HD is known to cause anticipation, a phenomenon in which subsequent generations show either a decreasing age of onset or an increasing severity of the disease. When the mutant gene is passed down through the father, anticipation occurs far more frequently. The CAG repeats’ instability during spermatogenesis is the cause of the anticipatory phenomenon [17]. Paternal transmission is nearly the only way that large expansions occur. Although they can occasionally inherit it from their mothers, children with juvenile-onset illness typically receive the enlarged allele from their fathers [18].

## Conclusions

This case series highlighted the clinical heterogeneity of HD and the wide range of expression of anticipation, even among families with identical CAG repeat expansions. While anticipation was evident in two families, it was absent in one, illustrating the complexity between genotype and phenotype interplay, along with the environmental factors that influence disease onset, manifestation, and progression. Our findings highlight the importance of detailed family history, clinical examinations, high suspicion, early genetic testing, and appropriate counseling to guide diagnosis and holistic support for affected individuals and relatives. As no curative treatment currently exists, timely recognition and multidisciplinary care remain the cornerstone of management for HD.
